# xCT Inhibition Increases Sensitivity to Vorinostat in a ROS-Dependent Manner

**DOI:** 10.3390/cancers12040827

**Published:** 2020-03-30

**Authors:** Keiko Miyamoto, Motoki Watanabe, Shogen Boku, Mamiko Sukeno, Mie Morita, Haruhito Kondo, Koichi Sakaguchi, Tetsuya Taguchi, Toshiyuki Sakai

**Affiliations:** 1 Department of Molecular-Targeting Prevention, Kyoto Prefectural University of Medicine, Kyoto 602-8566, Japan; keiko-m@koto.kpu-m.ac.jp (K.M.); bokusho@koto.kpu-m.ac.jp (S.B.); kondou95@gmail.com (H.K.); 2 Department of Endocrine and Breast Surgery, Kyoto Prefectural University of Medicine, Kyoto 602-8566, Japan; ksak@koto.kpu-m.ac.jp (K.S.); ttaguchi@koto.kpu-m.ac.jp (T.T.); 3 Drug Discovery Center, Kyoto Prefectural University of Medicine, Kyoto 602-8566, Japan; sukeno@koto.kpu-m.ac.jp (M.S.); miemori@koto.kpu-m.ac.jp (M.M.); tsakai@koto.kpu-m.ac.jp (T.S.)

**Keywords:** vorinostat, HDAC inhibitor, glutathione, ROS, xCT, salazosulfapyridine, ferroptosis

## Abstract

As histone deacetylase inhibitors (HDACIs) have limited efficacy against solid tumors, we investigated whether and how oxidative stress is involved in sensitivity to HDACIs to develop a novel therapeutic option of HDACIs treatment. We first tested whether a reduction of the antioxidant glutathione (GSH) by glutamine deprivation affects sensitivity to a commercially available HDACI vorinostat and reactive oxygen species (ROS) accumulation. Next we investigated the relationship between a glutamate-cystine transporter xCT and the efficacy of vorinostat using siRNA of xCT and bioinformatic analyses. Finally, we verified the combinatory effects of vorinostat and the xCT inhibitor salazosulfapyridine (SASP) on ROS accumulation, cell death induction, and colony formation. Glutamine deprivation increased vorinostat-mediated cell death with ROS accumulation. Genetic ablation of xCT improved the efficacy of vorinostat, consistent with the results of public data analyses demonstrating that xCT expressions positively correlate with insensitivity to HDACIs in many types of cancer cell lines. Vorinostat caused ROS accumulation when combined with SASP, possibly resulting in synergistic ferroptosis. Our study provides a novel mechanistic insight into the mechanism underlying sensitivity to HDACIs involving xCT, suggesting xCT to be a promising predictive marker of HDACIs and rationalizing combinatory therapy of HDACIs with xCT inhibitors to induce ferroptosis.

## 1. Introduction

Histone deacetylase inhibitors (HDACIs) were expected to be effective for cancer treatment because they exhibited a variety of antitumor effects, such as cell cycle arrest, promotion of differentiation, and induction of cell death [1,2,3]. In particular, suberoylanilide hydroxamic acid, also known as vorinostat, was the first HDACI approved by the Food and Drug Administration for the treatment of cutaneous T cell lymphoma; however, vorinostat was less effective against solid tumors than expected in clinical studies [4,5,6,7] because of its adverse effects [7,8] and/or the development of resistant cells [9,10].

As HDACIs increase the generation of reactive oxygen species (ROS) to lead to cell death [1,3,11], the mechanisms of oxidative stress induction may be involved in resistance to HDACIs. Indeed, the expression levels of thioredoxin, which is a major reducing protein that functions as a ROS scavenger [12], was related to resistance to ROS-inducing anticancer therapies [13,14,15], including HDACIs treatment [14,16]. In other studies, HDACI-resistant cells had increased levels of intracellular glutathione (GSH), which is the most abundant antioxidant [17].

xCT (coded by the *SLC7A11* gene), a light chain subunit of the glutamate-cystine antiporter system Xc(-), mediates cellular uptake of cystine in exchange for glutamate [18,19]. Cystine is rapidly reduced to cysteine, which is a rate-limiting precursor for GSH. Therefore, xCT is essential for maintaining the level of intracellular GSH and the redox balance. Recently, xCT inhibition was suggested as a potential therapeutic strategy for cancer due to the depletion of GSH and induction of ferroptosis, which is an iron- and ROS-dependent form of regulated cell death [20,21,22,23]. A potent xCT inhibitor, salazosulfapyridine (SASP), which is currently available for the treatment of inflammatory bowel disease and rheumatoid arthritis, has been considered to be repurposed as a promising antitumor drug [24,25,26,27,28,29,30,31,32].

In the present study, we first investigated the roles of glutamine and GSH in vorinostat-induced cell death and ROS accumulation. Furthermore, we examined whether genetic ablation and pharmacological inhibition of xCT can increase sensitivity to vorinostat by altering the cellular redox status. Lastly, we found that SASP-mediated ferroptosis may be promoted by HDAC inhibition. Thus, our study demonstrated a previously unknown mechanism of vorinostat sensitivity that is involved in xCT expression, in addition to suggesting the feasible combination of xCT inhibitors with vorinostat to improve its efficacy in anticancer treatment.

## 2. Results

### 2.1. Glutamine Deprivation Increases Sensitivity to Vorinostat

**Figure 1:**
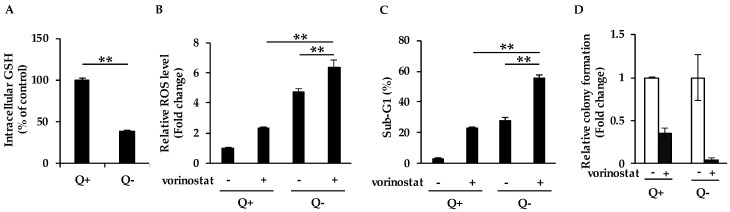
Glutamine deprivation increases sensitivity to vorinostat. (**A**) Measurement of intracellular glutathione (GSH) levels in media with/without glutamine. MDA-MB-231 cells were cultured in the presence of 4 mM glutamine (Q+) or in the absence of glutamine (Q−) for 24 h and subjected to the intracellular GSH content assay. The data obtained with complete media were taken as 100%. Columns, means (*n* = 3); bars, SD. ** *p* < 0.01. (**B**) Flow cytometry analysis of ROS levels in cells treated with vorinostat in media with/without glutamine. MDA-MB-231 cells were cultured in the presence of 4 mM glutamine (Q+) or in the absence of glutamine (Q−) for 24 h, and then treated with 1.6 μM vorinostat for 48 h. The amount of ROS in cells was detected by CM-H2DCFDA using a flow cytometer. The data obtained with dimethyl sulfoxide (DMSO) control in complete media were taken as 1. Columns, means (*n* = 3); bars, SD. ** *p* < 0.01. (**C**) Flow cytometry analysis of cell death after vorinostat treatment in media with/without glutamine. MDA-MB-231 cells were cultured in the presence of 4 mM glutamine (Q+) or in the absence of glutamine (Q−) for 24 h, and then treated with 1.6 μM vorinostat for 144 h. The percentages of cells in the sub-G1 population were analyzed by flow cytometry. Columns, means (*n* = 3); bars, SD. ** *p* < 0.01. (**D**) The colony formation of cells treated with vorinostat in media with/without glutamine. MDA-MB-231 cells were cultured in the presence of 4 mM glutamine (Q+) or in the absence of glutamine (Q−) for 24 h, and then treated with 1.6 μM vorinostat for 144 h. Colony formation was quantified using ImageJ. The data obtained with DMSO control in each condition were taken as 1. Columns, means (*n* = 3); bars, SD.

As glutamine in media is thought to be a source of intracellular glutathione (GSH), we first incubated cells with/without glutamine to examine whether glutamine deprivation affects intracellular GSH content. As expected, the deprivation of glutamine led to a significant decrease in the intracellular GSH content in human breast cancer MDA-MB-231 cells (Figure 1A). Next, we analyzed whether the deprivation of glutamine affects ROS accumulation in cells treated with vorinostat at 1.6 μM, which is close to IC50 values determined after 72 h exposure in both MDA-MB-231 and human colon cancer HCT116 cells (Supplementary Figure S1A), and Cmax values (1.2 ± 0.62 μM). We observed that the deprivation of glutamine increased vorinostat-mediated intracellular ROS accumulation (Figure 1B), suggesting that vorinostat was able to induce ROS due to the reduction of the antioxidant GSH. Consistent with these results, vorinostat induced more marked cell death (Figure 1C) and further suppressed colony formation (Figure 1D) in the absence of glutamine than in the complete media. Taken together, glutamine deprivation led to the reduction of the intracellular GSH level, which increased sensitivity to vorinostat with ROS accumulation.

### 2.2. Genetic Ablation of xCT Increases Sensitivity to Vorinostat with ROS Accumulation

**Figure 2:**
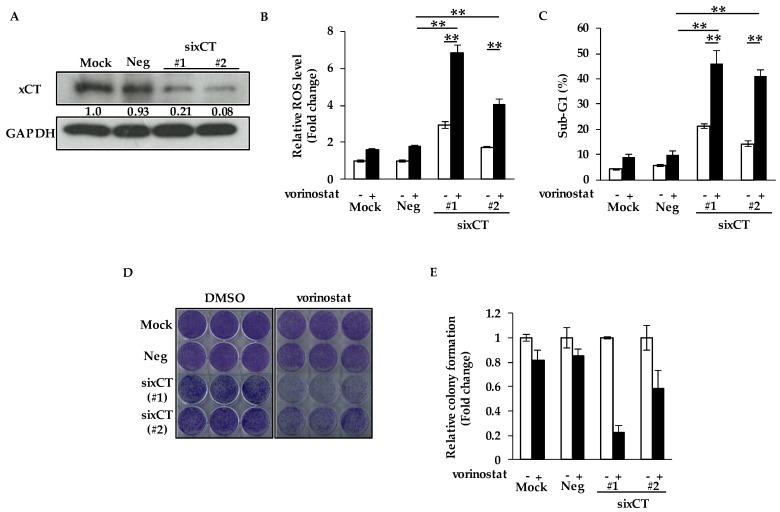
Genetic ablation of xCT increases sensitivity to vorinostat with reactive oxygen species (ROS) accumulation. (**A**) The knockdown efficacies of sixCT were validated by Western blotting in MDA-MB-231 cells. The gray value of bands was assessed by imageJ. GAPDH was used as a loading control. (**B**) Flow cytometry analysis of ROS levels in cells treated with vorinostat after transfection of sixCT or negative control siRNA (siNeg). MDA-MB-231 cells were treated with 1.6 μM vorinostat for 48 h following the treatment of each siRNA. The amount of ROS in cells was detected by CM-H2DCFDA using a flow cytometer. The data obtained with DMSO control in siNeg were taken as 1. Columns, means (*n* = 3); bars, SD. ** *p* < 0.01. (**C**) Flow cytometry analysis of cell death after vorinostat treatment with/without transfection of sixCT or siNeg. MDA-MB-231 cells were treated with 1.6 μM vorinostat for 144 h following the treatment of each siRNA. The percentages of cells in the sub-G1 population were analyzed by flow cytometry. Columns, means (*n* = 3); bars, SD. ** *p* < 0.01. (**D,E**) Colony formation of cells treated with vorinostat after transfection of sixCT or siNeg. MDA-MB-231 cells were treated with 1.6 μM vorinostat for 144 h following the treatment of each siRNA, and then the media were replaced. After the cells were cultured for 3–7 more days, the colonies were stained with crystal violet. Representative images of colony formation (*n* = 3) are shown (**D**). Colony formation was quantified using ImageJ (**E**). The data obtained with DMSO control for each siRNA were taken as 1. Columns, means (*n* = 3); bars, SD.

Intracellular GSH levels are known to be maintained by the expression of xCT, which promotes the uptake of cystine at the cell membrane. In order to examine whether the depletion of xCT affects sensitivity to vorinostat, we knocked down xCT using two siRNAs targeting different sequences of the SLC7A11 gene encoding xCT. We confirmed sufficient knockdown efficacies of xCT in MDA-MB-231 cells by Western blotting (Figure 2A).

We then assessed ROS levels in xCT-depleted cells by flow cytometry. As shown in Figure 2B, the depletion of xCT increased vorinostat-mediated ROS levels. We next found that the depletion of xCT significantly increased vorinostat-induced cell death, although vorinostat slightly induced cell death in cells treated with negative control siRNA (siNeg) (Figure 2C). Consistent with these results, vorinostat markedly suppressed colony formation by cells treated with sixCT, although vorinostat only slightly suppressed that by cells treated with siNeg (Figure 2D,E). Therefore, sensitivity to vorinostat was increased by depleting xCT.

### 2.3. The Expression of xCT is Modestly but Significantly Correlated with Sensitivity to HDACIs

**Figure 3:**
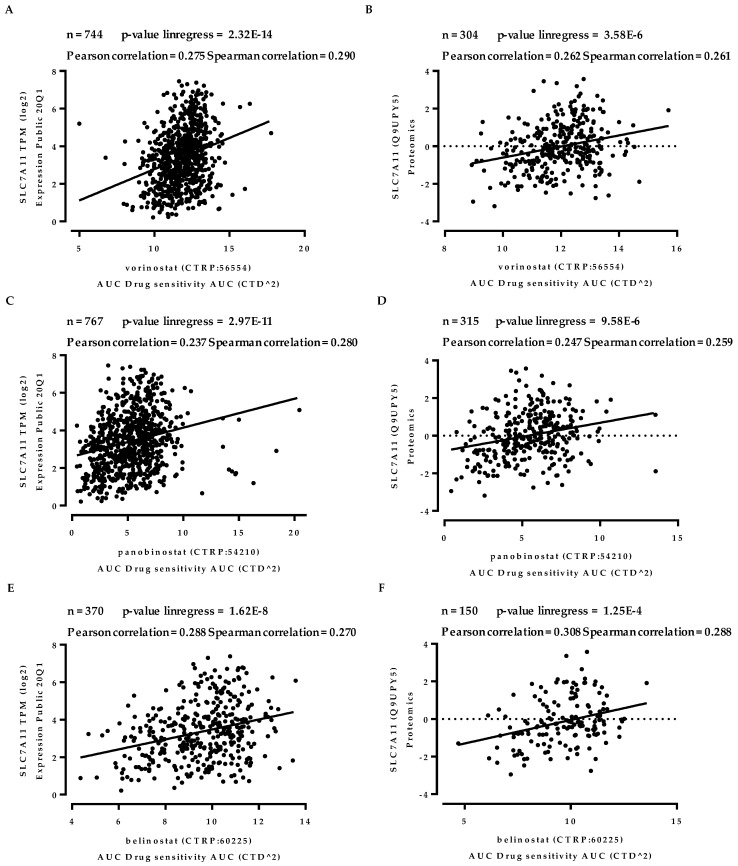
The expression of xCT is modestly but significantly correlated with sensitivity to Histone deacetylase inhibitors (HDACIs). Scatter plots depicting correlation of xCT expression and sensitivity to HDACIs using DepMap analysis. *X*-axis: the area-under-curve (AUC) of vorinostat (**A**,**B**), panobinostat (**C**,**D**), and belinostat (**E**,**F**) in pan-cancer cell lines. *Y*-axis: gene expressions of the *SLC7A11* gene (coding xCT) normalized transcripts per million, log scale (**A**,**C**,**E**) and protein expressions of that, log scale (**B**,**D**,**F**).

Next, we evaluated the correlation between xCT expression and sensitivity to HDACIs using the Cancer Dependency Map (DepMap) project (https://depmap.org/portal/) conducted by the Broad Institute [33,34]. A significant positive correlation was observed between the gene expression of SLC7A11 and insensitivity to vorinostat in a panel of 744 cancer cell lines (Pearson correlation = 0.275, Spearman correlation score = 0.290, *p* < 0.001) (Figure 3A).

Similar results were obtained with the protein expression of xCT and insensitivity to vorinostat in a panel of 304 cancer cell lines (Pearson correlation = 0.262, Spearman correlation score = 0.261, *p* < 0.001) (Figure 3B). Furthermore, insensitivity to other HDACIs, such as panobinostat (Figure 3C,D) and belinostat (Figure 3E,F), was modestly but significantly correlated with gene and protein expressions of xCT in a panel of hundreds of cancer cell lines. These results suggest that the expression levels of xCT are involved in sensitivity to HDACIs.

### 2.4. SASP Increases Sensitivity to Vorinostat by Reducing Intracellular GSH Levels and ROS Accumulation

**Figure 4:**
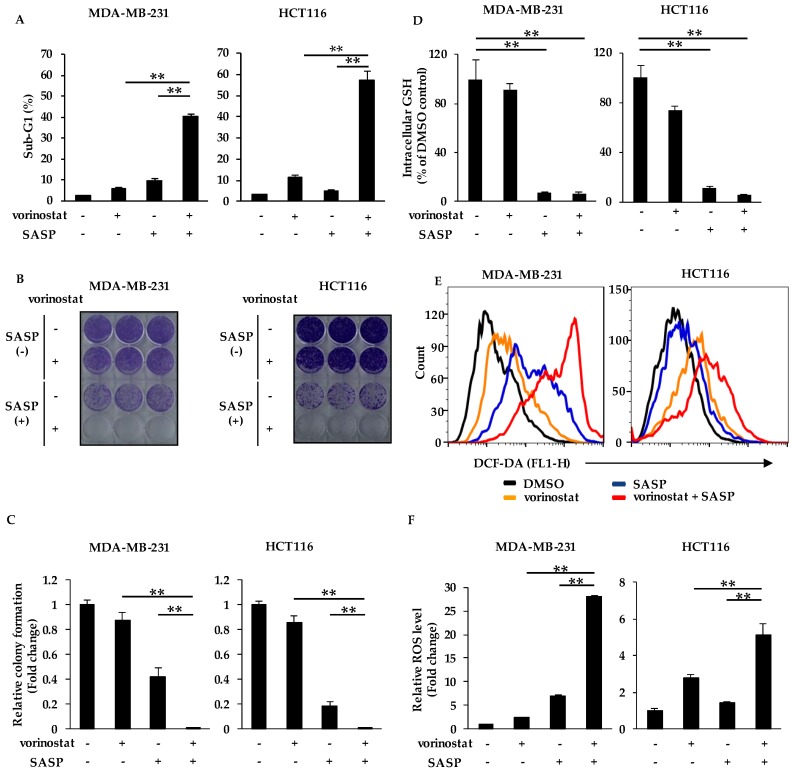
Salazosulfapyridine (SASP) increases sensitivity to vorinostat by reducing intracellular GSH levels and ROS accumulation. (**A**) Flow cytometry analysis of cell death induced by cotreatment of vorinostat with SASP. MDA-MB-231 and HCT116 cells were treated with 1.6 μM vorinostat, 500 μM SASP, or a combination of both for 144 h. The percentages of cells in the sub-G1 population were analyzed by flow cytometry. Columns, means (*n* = 3); bars, SD. ** *p* < 0.01. (**B**,**C**) Colony formation of cells cotreated with vorinostat and SASP. MDA-MB-231 and HCT116 cells were treated with 1.6 μM vorinostat, 500 μM SASP, or a combination of both for 144 h, and then the media were replaced. After the cells were cultured for 3–7 more days, the colonies were stained with crystal violet. Representative images of colony formation (*n* = 3) are shown (**B**). Colony formation was quantified using ImageJ (**C**). The data obtained with DMSO control were taken as 1. Columns, means (*n* = 3); bars, SD. ** *p* < 0.01. (**D**) Measurement of intracellular glutathione (GSH) levels in cells cotreated with vorinostat and SASP. MDA-MB-231 and HCT116 cells were treated with 1.6 μM vorinostat, 500 μM SASP, or a combination of both for 24 h, and then subjected to the intracellular GSH content assay. The data obtained with DMSO control were taken as 100%. Columns, means (*n* = 3); bars, SD. ** *p* < 0.01. (**E**,**F**) Flow cytometry analysis of ROS levels in cells cotreated with vorinostat and SASP. MDA-MB-231 and HCT116 cells were treated with 1.6 μM vorinostat, 500 μM SASP, or a combination of both for 48 h. Representative histogram patterns of ROS levels are shown (**E**). The amount of ROS in cells was detected by CM-H2DCFDA using a flow cytometer (**F**). The data obtained with DMSO control were taken as 1. Columns, means (*n* = 3); bars, SD. ** *p* < 0.01.

Based on our finding that the xCT inhibition can increase sensitivity to vorinostat, we hypothesized that the combination of vorinostat with salazosulfapyridine (SASP), which is a specific inhibitor of xCT, can induce synergistic cell death. To test this, we examined the sub-G1 population by flow cytometry in MDA-MB-231 and HCT116 cells after treatment with vorinostat and/or SASP for 144 h. Vorinostat induced marked cell death in both cell lines when cotreated with SASP at 500 μM (Figure 4A), which was close or below to IC50 values in MDA-MB-231 and HCT116 cells (Supplementary Figure S1B).

We then performed a colony formation assay using MDA-MB-231 and HCT116 cells treated with vorinostat and/or SASP, and confirmed that their combination markedly suppressed colony formation (Figure 4B,C). Indeed, SASP reduced intracellular levels of GSH (Figure 4D), and significant ROS accumulation was observed by the combined treatment of vorinostat with SASP in both cell lines (Figure 4E,F). These results suggest that SASP increases sensitivity to vorinostat via ROS accumulation in the same manner as the genetic knockdown of xCT using siRNA.

### 2.5. The Combined Effects of Vorinostat with SASP are Dependent on GSH and ROS

**Figure 5:**
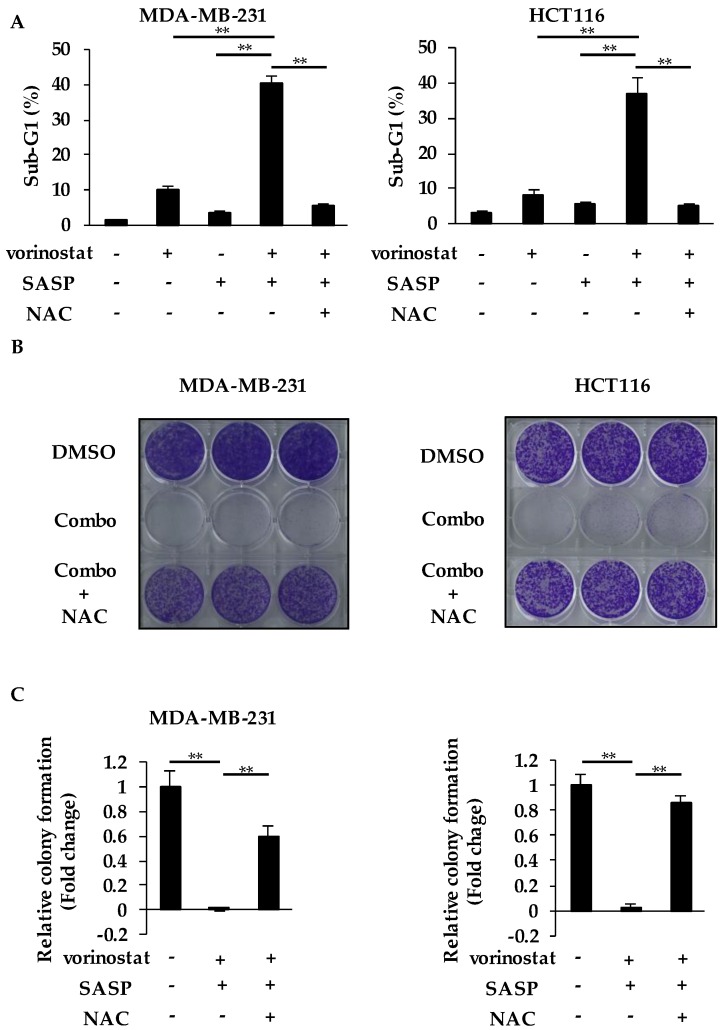
The combined effects of vorinostat with SASP are dependent on GSH and ROS. (**A**) Flow cytometry analysis of cell death induced by cotreatment of vorinostat with salazosulfapyridine (SASP) in the presence or absence of N-acetylcysteine (NAC). MDA-MB-231 and HCT116 cells were treated with 1.6 μM vorinostat, 500 μM SASP, or a combination of both with/without 5 mM NAC for 144 h. The percentages of cells in the sub-G1 population were analyzed by flow cytometry. Columns, means (*n* = 3); bars, SD. ** *p* < 0.01. (**B**,**C**) Colony formation of cells cotreated with vorinostat and SASP in the presence or absence of NAC. MDA-MB-231 and HCT116 cells were treated with a combination of 1.6 μM vorinostat and 500 μM SASP with/without 5 mM NAC for 144 h, and then the media were replaced. After the cells were cultured for 3–7 more days, the colonies were stained with crystal violet. Representative images of colony formation (*n* = 3) are shown (**B**). Colony formation was quantified using ImageJ (**C**). The data obtained with DMSO control were taken as 1. Columns, means (*n* = 3); bars, SD. ** *p* < 0.01.

We performed the add-back experiment using N-acetyl-L-cysteine (NAC), which is a precursor of GSH, to examine whether the accumulated ROS with reduced GSH played a role in the combined effects of vorinostat with SASP. As shown in Figure 5A, the supplementation of NAC completely reversed the cell death induced by the combination treatment to the basal level in both cell lines.

Consistent with this, the suppression of colony formation by the combination was also reversed by the supplementation of NAC (Figure 5B,C). Taken together, the combined effects of vorinostat with SASP depend on the accumulation of ROS caused by a decrease in intracellular GSH levels, possibly due to SASP-mediated inhibition of xCT.

### 2.6. The Combinatory Treatment of Vorinostat with SASP May Induce Ferroptosis

**Figure 6:**
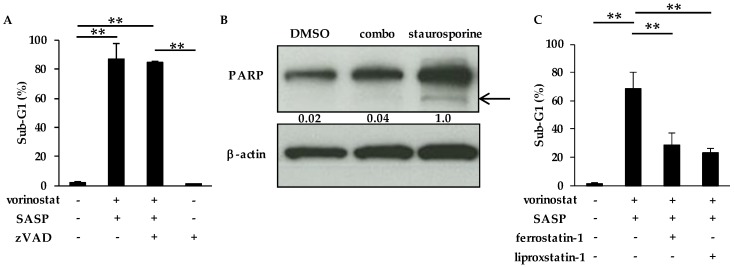
The combinatory treatment of vorinostat with SASP may induce ferroptosis. (**A**) Flow cytometry analysis of cell death induced by cotreatment of vorinostat with salazosulfapyridine (SASP) in the presence or absence of the pan caspase inhibitor zVAD-fmk. MDA-MB-231 cells were treated with the combination of 1.6 μM vorinostat and 500 μM SASP with/without 20 μM zVAD-fmk for 144 h. The percentages of cells in the sub-G1 population were analyzed by flow cytometry. Columns, means (*n* = 3); bars, SD. ** *p* < 0.01. (**B**) The cleavage of PARP (poly (ADP-ribose) polymerase) after combined treatment with vorinostat and SASP. MDA-MB-231 cells were treated with 1.6 μM vorinostat and 500 μM SASP for 144 h, and then treated with 1 μM staurosporine (as a positive control of apoptosis induction) for 6 h. Cleaved PARP was analyzed by Western blotting. The gray value of bands was assessed by imageJ. β-Actin was used as a loading control. (**C**) Flow cytometry analysis of cell death induced by cotreatment of vorinostat with SASP in the presence or absence of the ferroptosis inhibitor ferrostatin-1 or liproxstatin-1. MDA-MB-231 cells were treated with the combination of 1.6 μM vorinostat and 500 μM SASP with/without 2 μM ferrostatin-1 or 0.5 μM liproxstatin-1 for 144 h. The percentages of cells in the sub-G1 population were analyzed by flow cytometry. Columns, means (*n* = 3); bars, SD. ** *p* < 0.01.

We next characterized the cell death induced by vorinostat and SASP. First, the addition of the pan-caspase inhibitor zVAD-fmk did not reverse the synergistic induction of cell death induced by combined vorinostat and SASP in MDA-MB-231 cells (Figure 6A).

Moreover, the cleavage of PARP (poly (ADP-ribose) polymerase) was not induced after the combination treatment (Figure 6B), suggesting that cotreatment of vorinostat with SASP did not induce caspase-dependent apoptosis. We then considered ferroptosis because SASP is known to be a ferroptosis inducer [20,21,22,23]. As expected, both ferrostatin-1 [35,36] and liproxstatin-1 [35], known ferroptosis inhibitors, attenuated cell death induced by the combination of vorinostat and SASP (Figure 6C), suggesting that combined vorinostat and SASP induced ferroptosis.

## 3. Discussion

Histone deacetylase inhibitors (HDACIs), which exhibit various anticancer effects, including cell death induction, have been expected as anticancer agents; however, they have only modest clinical activity especially against solid tumors due to complicated resistance mechanisms. In the present study, we found a novel mechanism of resistance to cell death induced by a commercially available HDACI vorinostat, which was involved in xCT-mediated ROS scavenging. Furthermore, cotreatment of vorinostat with xCT inhibition, including salazosulfapyridine (SASP) treatment, resulted in the accumulation of ROS and induction of synergistic cell death, which was supposed to be an iron-dependent form of nonapoptotic cell death: ferroptosis.

As appropriate predictive markers for HDACIs are urgently needed to improve their therapeutic effects in clinical settings, many markers of resistance to HDACIs treatment have been proposed: the nuclear accumulation of signal transducers, activator of transcription (STAT)1 and phosphorylated STAT3 [37], overexpression of HR23B [38,39], and low expression of HDAC6 [39] and Fbw7 [40]. Of note, the expression of ROS scavengers, such as thioredoxin [16], catalase, and peroxisomal protein PEX3 [41], has also been reported to be involved in resistance to vorinostat. Considering our observations that the depletion of xCT overcame the resistance to vorinostat-induced cell death (Figure 2C) and xCT expressions are inversely correlated with the susceptibility to HDACIs (Figure 3), xCT levels may serve as a predictive marker to stratify patients unresponsive to HDACIs treatment.

Although ROS accumulation is important for HDACIs-mediated antitumor efficacy [1,3,11], ROS detoxification is increased in cancer [42,43,44], which causes resistance to HDACIs-induced cell death. In particular, the glutathione (GSH)-related ROS detoxification system is known to cause resistance to several antitumor agents that induce ROS. Therefore, targeting the GSH-related ROS detoxification system may be reasonable to overcome resistance to HDACIs-induced cell death. Consistent with this idea, β-phenylethyl isothiocyanate, which is a natural compound with the ability to deplete intracellular GSH [45,46], was reported to increase vorinostat-induced apoptosis of human leukemia cells [17]. In our study, the approved oral drug SASP was repurposed to inhibit xCT and reduce intracelluar GSH levels as a sensitizer to HDACIs in cancer treatment.

We here discuss the feasibility of the combination of vorinostat and SASP in the clinic. First, vorinostat has been considered to cause an accumulation of ROS in transformed but not normal cells, since vorinostat induces the reducing protein thioredoxin mainly in normal cells but not in transformed cells [16]. Considering that we treated cells with vorinostat at 1.6 μM (close to Cmax values: 1.2 ± 0.62 μM) in our experiments, vorinostat may not largely affect normal cells in this condition. Next, SASP is already in early-phase studies as a specific xCT inhibitor [30,32]; however, the pharmacokinetics of SASP appears to fluctuate in the physiological conditions of the body. Cmax values of SASP were observed in a wide range of concentrations in the clinical studies (approximately 6.5–120 μg/mL: 16–300 μM) [30,32], possible because SASP is metabolized by intestinal bacteria to 5-aminosalicylic acid and sulfapyridine, which lose the activity of xCT inhibition. Therefore, water-soluble form of SASP, which has been currently developed to be stably absorbed and maintain the blood concentration [32], may be more safely used together with HDACIs.

Other novel finding taken from our study is that the combinatorial treatment of vorinostat with SASP may induce ferroptosis (Figure 6). Although SASP is a known major ferroptosis inducer [20,21,22,23], SASP treatment alone caused only limited cell death (Figure 4A) and ROS accumulation (Figure 4F), suggesting that SASP-induced ferroptosis requires HDAC inhibition at least in our experimental setting. Recently, glutathione peroxidase 4 (GPX4) was reported to be a key enzyme that negatively regulates xCT-GSH-dependent ferroptosis by suppressing lipid ROS [47,48,49]. GPX4 is stabilized by the chaperone protein HSP90 [50], whose acetylation impairs chaperone activity and leads to degradation of its client proteins [51]. Therefore, vorinostat may degrade GPX4 protein via HSP90 acetylation, resulting in SASP-induced ferroptosis with ROS accumulation, although this is a hypothesis to be tested in the future.

## 4. Materials and Methods

### 4.1. Reagents

Vorinostat (suberoylanilide hydroxamic acid, SAHA) was purchased from Cayman Chemical (Ann Arbor, MI, USA). Salazosulfapyridine (SASP), ferrostatin-1, and liproxstatin-1 were purchased from Sigma-Aldrich (St. Louis, MO, USA). N-acetyl-L-cysteine (NAC) was purchased from Nacalai tesque (Japan). zVAD-fmk was purchased from R&D systems (Minneapolis, MN, USA). Staurosporine was purchased from abcam (Cambridge, UK). Vorinostat, SASP, ferrostatin-1, liproxstatin-1, zVAD-fmk, and staurosporine were dissolved in the solvent dimethyl sulfoxide (DMSO) to make stock solutions. DMSO was used at the final concentration of 0.5% or lower in all experiments without any effect on the phenomenon compared with non-DMSO treated controls. NAC was dissolved in Milli-Q water.

### 4.2. Cell Culture

The human breast cancer MDA-MB-231 cells and the human colon cancer HCT116 cells were obtained as the cell lines of NCI-60 from the NCI Developmental Therapeutics Program. The cell lines were used within 2–3 months after cell recovery and tested for mycoplasma contamination using the MycoAlertTM Mycoplasma Detection Kit (Lonza, Basel, Switzerland). MDA-MB-231 cells were cultured in RPMI-1640 supplemented with 10% fetal bovine serum (FBS), 4 mM L-glutamine, 50 U/mL of penicillin, and 100 μg/mL of streptomycin. HCT116 cells were cultured in Dulbecco’s Modified Eagle’s Medium (DMEM) supplemented with 10% FBS, 4 mM L-glutamine, 50 U/mL of penicillin, and 100 μg/mL of streptomycin. The culture mediums were used within 2 weeks to avoid degradation of L-glutamine. All cells were incubated at 37 °C in a humidified atmosphere of 5% CO_2_.

### 4.3. Quantification of Intracellular Glutathione

Intracellular glutathione (GSH) was measured using the GSH-Glo^TM^ Glutathione assay kit (Promega, Madison, WI, USA) according to the manufacturer’s instructions. Briefly, 5000 cells per well were seeded in 96-well optical bottom plates (Thermo scientific, Waltham, MA, USA). After incubating for 24 h, cells were treated with each reagent for 24 h. After removing medium from each well, cells were incubated in 100 μL of GSH-Glo^TM^ reagent for 30 min, and then 100 μL of reconstituted Luciferin Detection Reagent for 15 min. Luminescent signals were measured using a microplate luminometer Centro LB 960 (Berthold Technologies GmbH & Co.KG, Germany).

### 4.4. Cell Viability Assay

Cell viability was measured by a Cell Counting Kit-8 assay (Dojindo, Japan) according to the manufacturer’s instructions. Cells were seeded in 96-well plates at a density of 2000 cells per well. After incubating for 24 h, cells were treated with each reagent for 72 h, and then the kit reagent WST-8 was added to the medium and incubated for 4 h. The absorbance at 450 nm of the samples was measured using SpectraMax iD5 (Molecular Devices, LLC, San Jose, CA, USA). All experiments shown were replicated at least twice.

### 4.5. Analysis of ROS Accumulation

The cells were seeded in 6-well plates at a density of 50,000 cells per well. After incubating for 24 h, cells were treated with each reagent for 48 h. Then cellular ROS content in 10,000 cells was measured by flow cytometry analysis using FACSCalibur (Becton Dickinson, Franklin Lakes, NJ, USA), followed by incubating the cells with CM-H2DCFDA (Invitrogen, Carlsbad, CA, USA) for 30 min. Cell Quest software (Becton Dickinson) was used to analyze the data. Histograms were created using Flowjo software (Tomy Digital Biology, Japan).

### 4.6. Analysis of Cell Death

Cells were treated with each agent for 144 h and harvested by trypsinization. After washing with PBS, the cells were suspended in PBS containing 0.1% Triton X-100, and the nuclei were stained with 25 μg/mL of propidium iodide. The DNA content in 10,000 cells was measured using FACSCalibur (Becton Dickinson). Cell Quest software (Becton Dickinson) was used to analyze the data. DNA fragmentation was quantified by the percentage of hypodiploid DNA as the sub-G1 population.

### 4.7. Colony Formation Assay

Cells were seeded at a density of 8000 cells per well in 6-well plates. After incubating for 24 h, cells were treated with each reagent for 144 h. Then the medium was replaced with fresh medium, and the cells were cultured for 3–7 more days. The colonies fixed in 10% formalin were stained by crystal violet. The area of stained colonies was quantified using the ImageJ program from the National Institutes of Health (Bethesda, MD, USA, https://imagej.nih.gov/ij/).

### 4.8. Small Interfering RNA Transfection

ON-TARGETplus siRNAs that targeted human SLC7A11 were obtained from Dharmacon (Lafayette, CO, USA). The following siRNAs were used: siSLC7A11 #1 (J-007612-10; ON-TARGETplus human SLC7A11 (23657) siRNA), CGGCAAACUUAUUGGGUCU; siSLC7A11 #2 (J-007612-11; ON-TARGETplus human SLC7A11 (23657) siRNA), AGGGUUAACAAGAGUAUAA; and negative control (D-001810-03-05; ON-TARGETplus Non-targeting siRNA), UGGUUUACAUGUUUUCUGA. After cells were seeded in 6-well plates, cells were transfected with 10 nM siRNA using Lipofectamine RNAiMAX (Invitrogen, Carlsbad, CA, USA) according to the manufacturer’s instructions. Twenty-four hours after transfection, the cells were treated with each agent for the indicated time.

### 4.9. Protein Isolation and Western Blotting

Cells were lysed in a buffer containing 50 mM Tris-HCl, 1% SDS, 1 mM DTT, and 0.43 mM 4-(2-aminoethyl) benzenesulfonyl fluoride hydrochloride. The lysates were sonicated and centrifuged at 20,400 *g* for 20 min at 4 °C, and the supernatant was collected. Equal amounts of the protein extract were subjected to SDS-PAGE, and transferred to a PVDF membrane (Millipore, Burlington, MA, USA). The following were used as the primary antibodies: anti-xCT rabbit antibody (#ab37185; abcam) and anti-GAPDH mouse antibody (HyTest, Turku, Finland). The signals were detected using a Chemi-Lumi One L (Nacalai Tesque) or an Immobilon Western Chemiluminescent HRP Substrate (Millipore). The band intensities of protein samples were quantified using ImageJ.

### 4.10. Public Data Acquisition

Datasets of the normalized area under the curve (AUC) values of each agent in each cell line and gene/protein expressions of SLC7A11 were obtained from the DepMap website (https://depmap.org/portal/). Pearson and Spearman’s correlation coefficients were used to evaluate the correlation between AUC values of each agent and SLC7A11 gene/protein expressions.

### 4.11. Statistical Analysis

All data are presented as the mean ± SD. The significance of differences of means between two groups was tested using the unpaired Student’s *t*-test, and that of comparisons between three or more groups was tested using one-way ANOVA with Dunnett’s post-hoc test. *p* < 0.05 was considered significant.

## 5. Conclusions

In summary, we demonstrated that the inhibition of xCT including SASP treatment may overcome resistance to vorinostat by accumulating ROS and inducing ferroptosis. Our findings have important implications not only for HDAC inhibition, which may be required to induce ferroptosis, but also for a feasible therapeutic avenue using SASP or other xCT inhibitors to expand the indications of vorinostat to solid tumors, although preclinical studies are needed.

## Supplementary Materials

The following are available online at https://www.mdpi.com/2072-6694/12/4/827/s1, Figure S1: Vorinostat and SASP inhibit cell growth in a dose-dependent manner, Figure S2: The uncropped Western blot bands used in this study.
