# Supplementary material for: xCT Inhibition Increases Sensitivity to Vorinostat in a ROS-Dependent Manner
Source: Cancers (Basel). 2020 Mar 30;12(4):827. doi: 10.3390/cancers12040827 (PMC7226257; doi:10.3390/cancers12040827)
Supplement: Supplementary file 1 [file cancers-12-00827-s001.pdf]

Supplementary Materials: xCT Inhibition Increases Sensitivity to Vorinostat in a ROS-Dependent Manner

Keiko Miyamoto, Motoki Watanabe, Shogen Boku, Mamiko Sukeno, Mie Morita, Haruhito Kondo, Koichi Sakaguchi, Tetsuya Taguchi and Toshiyuki Sakai

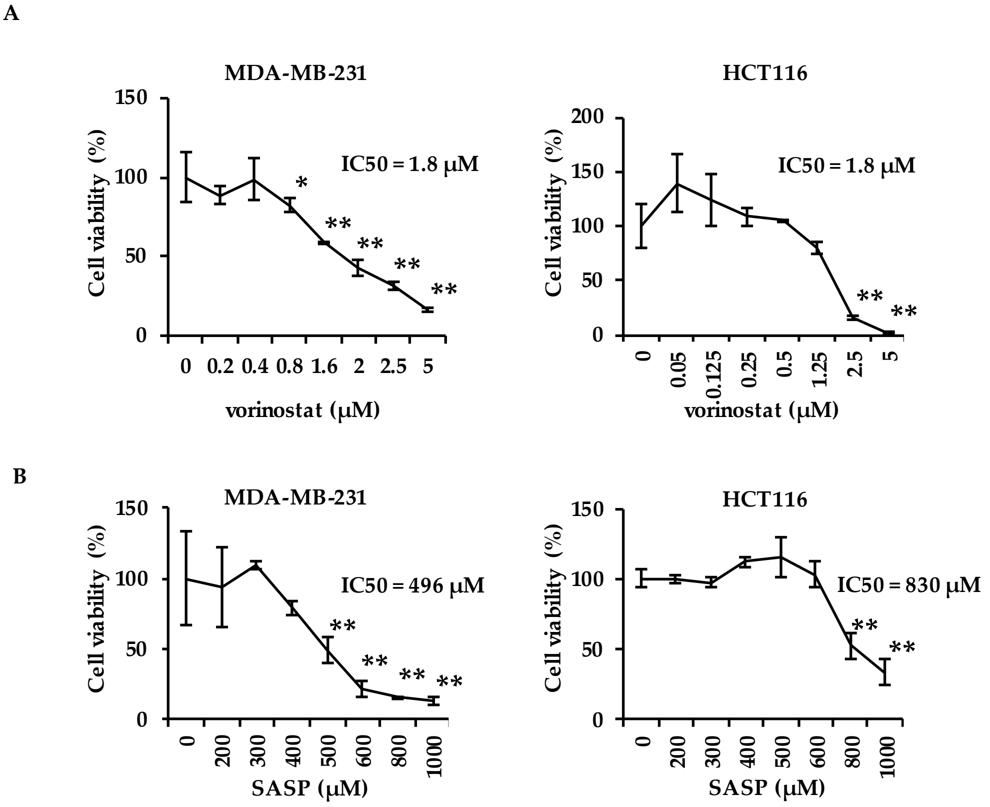

Figure S1. Vorinostat and SASP inhibit cell growth in a dose-dependent manner. (**A**) Growth inhibitory effect of vorinostat on cancer cells. MDA-MB-231 and HCT116 cells were treated with vorinostat at the indicated concentrations for 72 h, and cell viability was measured by a Cell Counting Kit-8 assay. The data obtained with DMSO control were taken as 100%. Columns, means (n = 3); bars, SD. * $p < 0.05$, ** $p < 0.01$. (**B**) Growth inhibitory effect of salazosulfapyridine (SASP) on cancer cells. MDA-MB-231 and HCT116 cells were treated with SASP at the indicated concentrations for 72 h, and cell viability was measured by a Cell Counting Kit-8 assay. The data obtained with DMSO control were taken as 100%. Columns, means (n = 3); bars, SD. ** $p < 0.01$. Statistical analyses were performed using one-way ANOVA with Dunnett's post-hoc test.

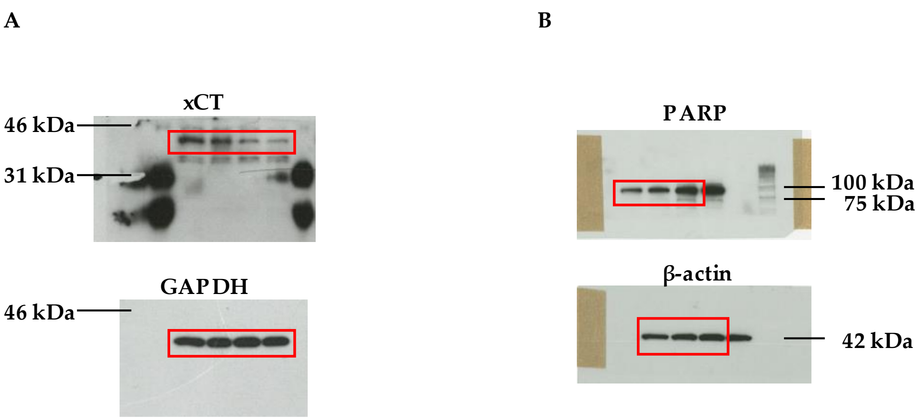

Figure S2. The uncropped Western blot bands used in this study. (**A**) The uncropped Western blot bands related to Figure 2A. (**B**) The uncropped Western blot bands related to Figure 6B.
